# Hepatocellular Carcinoma Recurrence after Hepatitis C Virus Therapy with Direct-Acting Antivirals. A Systematic Review and Meta-Analysis

**DOI:** 10.3390/jcm10081694

**Published:** 2021-04-15

**Authors:** Leonardo Frazzoni, Usama Sikandar, Flavio Metelli, Sinan Sadalla, Giuseppe Mazzella, Franco Bazzoli, Lorenzo Fuccio, Francesco Azzaroli

**Affiliations:** 1 IRCCS Azienda Ospedaliero-Universitaria di Bologna, 40138 Bologna, Italy; leonardo.frazzoni@unibo.it (L.F.); franco.bazzoli@unibo.it (F.B.); lorenzo.fuccio@unibo.it (L.F.); 2 Department of Medical and Surgical Sciences, Gastroenterology Unit, S. Orsola-Malpighi Hospital, University of Bologna, 40138 Bologna, Italy; usama.sikandar@studio.unibo.it (U.S.); flaviomet90@gmail.com (F.M.); sinan.sadalla@studio.unibo.it (S.S.); giuseppe.mazzella@unibo.it (G.M.)

**Keywords:** direct-acting antivirals (DAAs), hepatocellular carcinoma (HCC), hepatitis C virus (HCV)

## Abstract

Background: Hepatocellular carcinoma (HCC) is a major cause of morbidity and mortality among patients with cirrhosis. The risk of HCC recurrence after a complete response among patients treated with direct-acting antivirals (DAAs) has not been fully elucidated yet. Aim: To assess the risk of HCC recurrence after DAA therapy for hepatitis C virus (HCV). Methods: A systematic review across PubMed, Scopus and Scholar up to November 2020, including full-text studies that assessed the pattern of HCC recurrence after DAA therapy for HCV. Random-effect meta-analysis and univariable metaregression were applied to obtain pooled estimates for proportions and relative risk (RR) and variables influential for the outcome, respectively. Results: Thirty-one studies with 2957 patients were included. Overall, 30% (CI, 26–34%) of the patients with a history of HCC experienced HCC recurrence after DAA therapy, at mean time intervals ranging from 4 to 21 months. This result increased when going from European studies (23%, CI, 17–28%) to US studies (34%, CI, 30–38%), to Egyptian studies (37%, CI, 27–47%), and to Asian studies (33%, CI, 27–40%). Sixty-eight percent (CI, 45–91%) of recurrent HCCs developed within 6 months of follow-up since DAA treatment, among the eight studies providing stratified data. Among the studies providing head-to-head comparisons, the HCC recurrence risk was significantly lower after DAA therapy than IFN (RR, 0.64; CI, 0.51–0.81), and after DAA therapy than no intervention (RR, 0.68; CI, 0.49–0.94). Conclusions: The recurrence of HCC after DAA is not negligible, being higher soon after the end of treatment and among non-European countries. DAA therapy seems to reduce the risk of HCC recurrence compared to an IFN regimen and no intervention.

## 1. Introduction

Hepatocellular carcinoma (HCC) is among the most frequent causes of cancer-related death all over the world; hepatitis C virus (HCV) is recognized as the most common main etiological factor in Western countries [1]. The curative treatments for HCC encompass liver transplantation, resection, and ablation with radiofrequency [2]. These approaches yield satisfactory long-term survival; however, they are hampered by tumor recurrence rates as high as 50% at 5 years [3].

Antiviral therapy with direct-acting antivirals (DAA) has replaced interferon as the standard treatment regimen for HCV, yielding eradication rates higher than 90% [4]. It was reported that interferon-based treatment was associated with a decrease in HCC incidence and recurrence [5], even though the mechanistic linkage was speculative. Indeed, whether this effect was due to sustained viral responses (SVRs) or to interferon-associated antineoplastic effects remained unknown [6]. Therefore, researchers are trying to assess whether DAA therapy replicates the interferon-mediated effects on HCC incidence and recurrence. Studies have consistently demonstrated that DAA therapy improves liver fibrosis, portal hypertension and the occurrence of de novo HCC [7]. However, some evidence coming from observational studies has suggested that patients with a history of a curative treatment for HCC and chronic hepatitis C who underwent DAA therapy experienced an increased HCC recurrence rate [8]. Systematic reviews and meta-analyses have reported that DAA therapy does not seem to significantly influence the HCC recurrence rate [9]; however, they were based on a limited number of studies. Subsequently, after the publication of such analyses, new studies have provided further evidence for this important topic.

Thus, the primary aim of the present systematic review and meta-analysis was to assess the risk and pattern of HCC recurrence in patients treated with DAAs. The key secondary aim was to compare the HCC recurrence risk after DAA therapy to that after IFN and no intervention.

## 2. Methods

We followed the Preferred Reporting Items for Systematic Reviews and Meta-Analyses (PRISMA) recommendations (Table 1) [10] and rated the methodological quality of the included studies through the Newcastle–Ottawa scale [11]. The Newcastle–Ottawa scale categorizes the risk of bias for the included studies into a four-tier system (i.e., unknown, low, medium, and high).

### 2.1. Literature Search and Study Selection

A comprehensive literature search was independently performed by four investigators (L.F.; U.S.; F.M.; S.S.) up to November 2020 by querying PubMed, Scopus, and Scholar using a combination of controlled vocabulary, medical subject headings (MeSH) terms, and keywords including “hepatocellular ca*” or “liver cancer”, “hepatitis C virus” or “chronic hepatitis c”, and “direct-acting antivirals” or “interferon-free”. The PubMed search string was ((chronic hepatitis c(MeSH Terms) OR (hepatitis c virus(MeSH Terms))) AND ((hepatocellular cancer(MeSH Terms)) OR (hepatocellular carcinoma(MeSH Terms)) OR (liver cancer(MeSH Terms))) AND ((direct-acting antivirals) OR (interferon-free) OR (DAA)).

To be included in the systematic review, studies had to report HCC recurrence among patients treated with DAA. Prospective and retrospective studies, published in the English language, with no minimum sample size, were considered for inclusion. Studies published in abstract form were excluded. We included studies reporting complete response from surgical or local ablative therapies; we excluded studies that administered interferon-based regimens in conjunction with DAAs.

Titles and abstracts were first screened. Then, the authors evaluated the full texts of the potentially relevant screened articles, including those meeting the inclusion criteria. Disputes were resolved by collegial discussion. The reasons for excluding studies from the selection process were recorded.

### 2.2. Data Extraction

The same four authors who performed the search (L.F.; U.S.; F.M.; S.S.) extracted data from each included study on a pre-specified datasheet. The following data were extracted from each study: the study design and country, the numbers of centers involved, the study size, the rate of recurrent HCC, the mean follow-up, the mean duration between prior HCC curative treatment and DAA therapy, and patients’ baseline characteristics (i.e., the mean MELD score, mean number of previous HCCs, proportion of patients with >1 previous HCC, and mean size of previous HCC).

### 2.3. Statistical Analysis

The proportions of patients experiencing HCC recurrence, as well as the relative risk (RR) for HCC recurrence, were pooled by a random effects model, along with 95% confidence intervals (CIs). We quantified the statistical heterogeneity according to the inconsistency I^2^ statistic, considering it high when I^2^ > 50%. A continuity correction factor equal to 0.1 was applied when no HCC recurrence was detected. Potential sources of heterogeneity were investigated through subgroup meta-analytic models according to study design (i.e., retrospective vs. prospective, or monocentric vs. multicentric) and country (i.e., Europe vs. Asia vs. United States), as well as through univariable metaregression analyses including the study size, mean time elapsed from HCC curative treatment to DAA therapy, mean follow-up duration, mean size of previous HCC, proportion of patients with >1 previous HCC, and mean baseline alpha-fetoprotein (AFP) level. Continuous data reported as medians with interquartile ranges or ranges were converted into means through approximation formulae, in order to perform meta-analyses. Publication bias was evaluated throughout the visual inspection of funnel plots and by the regression test as proposed by Harbord, Egger and Sterne [12]. All the analyses were performed with R by using the metafor package [13].

## 3. Results

### 3.1. Study Characteristics

**Figure 1:**
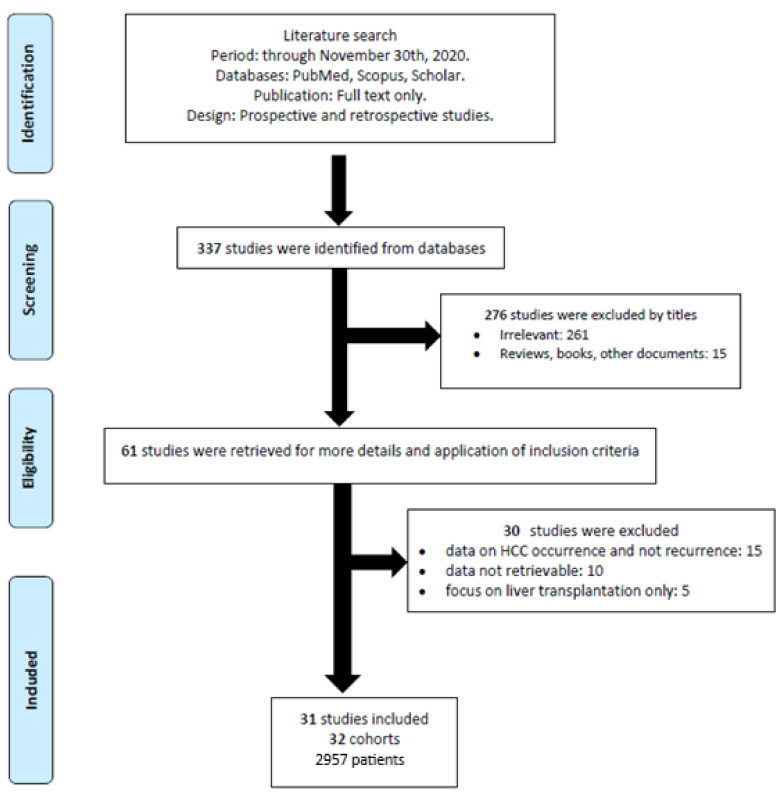
Flow-chart describing the process of study selection.

Overall, the search strategy identified 337 studies, of which 61 were considered for inclusion. After excluding 15 studies presenting data on HCC occurrence and not recurrence, 10 reporting incomplete data, and 5 focusing on post-transplantation HCC recurrence, 31 studies were finally included in the present systematic review [8,14,15,16,17,18,19,20,21,22,23,24,25,26,27,28,29,30,31,32,33,34,35,36,37,38,39,40,41,42,43] (Figure 1), encompassing 2957 patients and 32 cohorts, as the ANRS study included two patient groups [14].

Seven studies were prospective, whereas 15 studies were multicentric. Eleven studies were performed in Europe, whereas 14 were conducted in Asian countries, two in North America, three in Egypt, and one in Australia. All the studies were published between 2016 and 2020. The study sizes ranged from 8 to 304 patients. The mean follow-up durations to identify HCC recurrence ranged from 3 to 50 months. The study characteristics are detailed in Table 2.

### 3.2. HCC Recurrence

**Figure 2:**
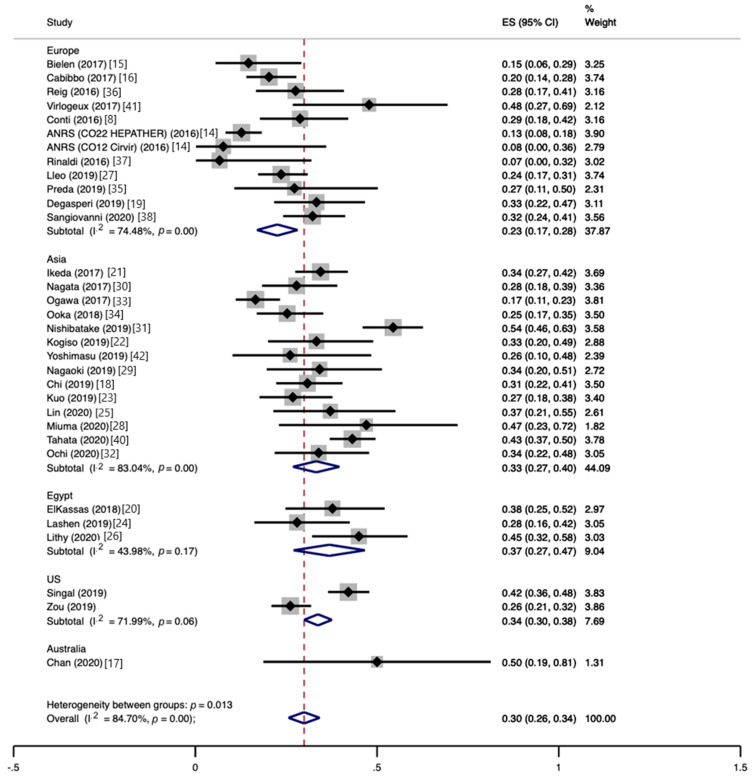
Proportion of patients developing hepatocellular carcinoma recurrence following direct-acting antiviral therapy.

The pooled HCC recurrence at the end of follow-up was 30% (CI, 26−34%), with high heterogeneity (I^2^ = 84.7%) (Figure 2). The proportion of patients experiencing HCC recurrence varied widely between studies, ranging from 7 to 54%, as did the mean time to recurrence, ranging from 4 to 21 months. According to subgroup analyses, the pooled HCC recurrence was similar between prospective and retrospective studies (28%, CI, 21−36%, vs. 30%, CI, 26−35%), whereas monocentric studies yielded significantly higher estimates than multicentric studies (36%, CI, 31−41%, vs. 25%, CI, 20−30%; *p* = 0.003). The recurrence of HCC was higher for Australian studies (50%, CI, 19−81%) than Egyptian studies (37%, CI, 27−47%), Asian studies (33%, CI, 27−40%), US studies (34%, CI, 30−38%), and European studies (23%, CI, 17−28%), with *p* = 0.013. No significant impact on HCC recurrence was found for the study size (*p* = 0.49), mean time elapsed from HCC curative treatment to DAA therapy (*p* = 0.32), mean follow-up duration (*p* = 0.58), mean size of previous HCC (*p* = 0.46), proportion of patients with >1 previous HCC (*p* = 0.92), or mean baseline alpha-fetoprotein (AFP) level (*p* = 0.50).

**Figure 3:**
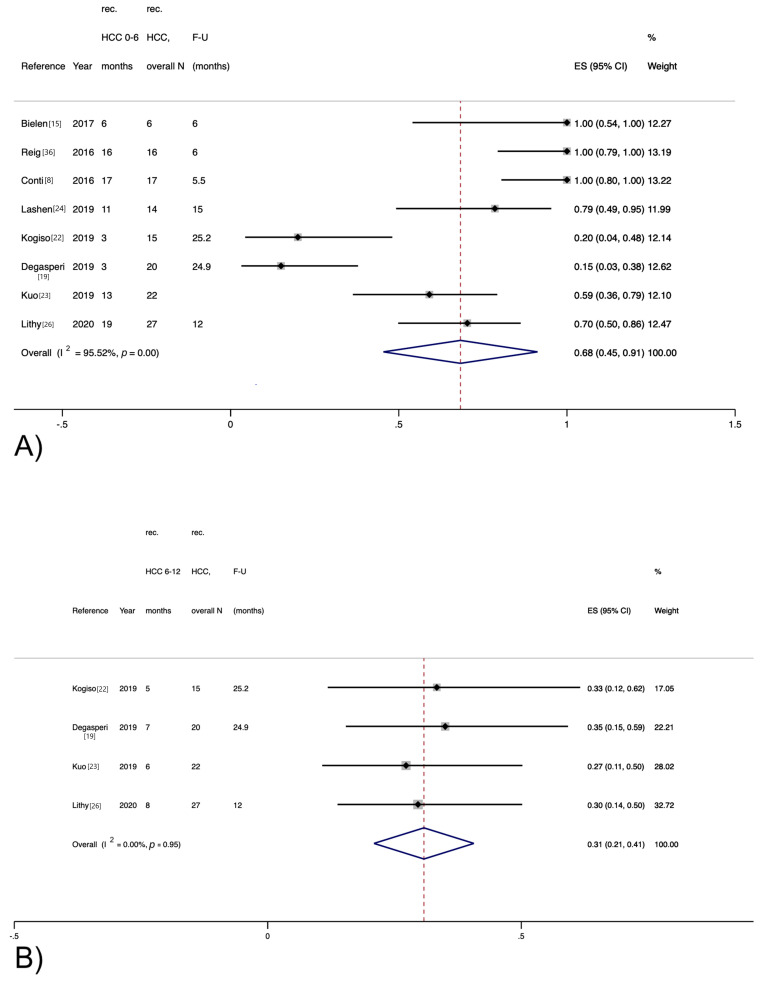
Recurrence rate for hepatocellular carcinoma stratified by time elapsed from direct-acting antiviral therapy. (**A**) Within 6 months; (**B**) Between 6 and 12 months.

Among the eight studies providing data on HCC recurrence stratified by the time elapsed from DAA therapy, 88 out of 137 (64%) recurrent HCCs developed within the first 6 months (Figure 3A), whereas 26 (19%) HCCs developed between 6 and 12 months, as reported by four studies (Figure 3B).

### 3.3. HCC Recurrence after DAA vs. IFN vs. No Intervention

**Figure 4:**
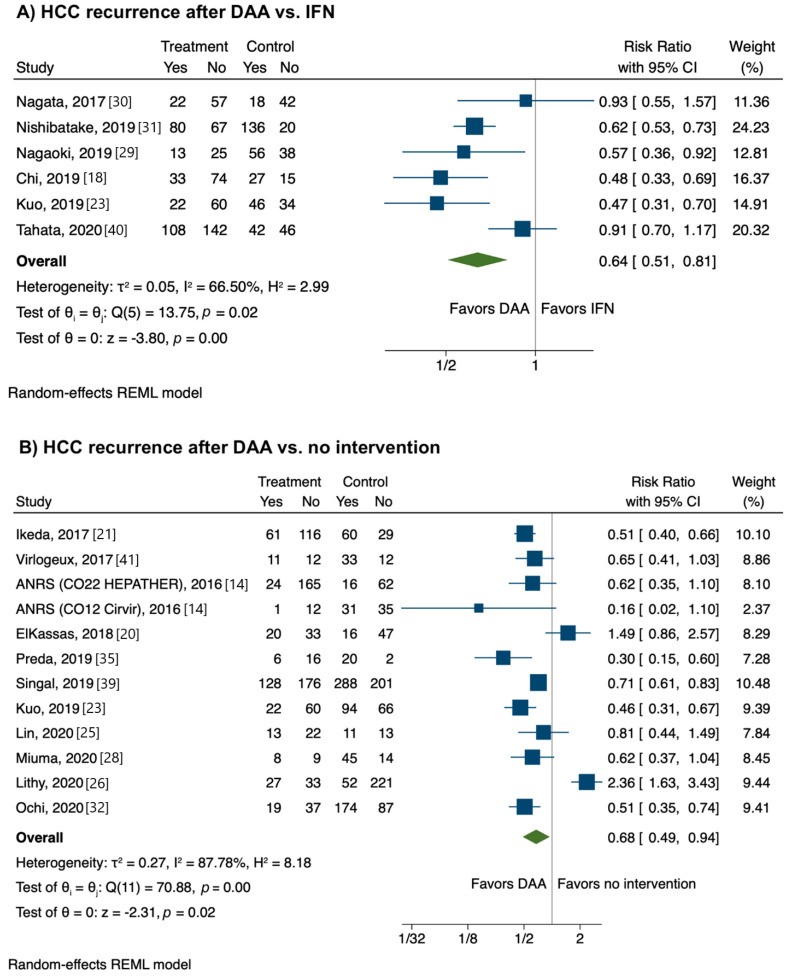
Relative risk of developing recurrent hepatocellular carcinoma according to strategy. (**A**) Direct-acting antivirals vs. interferon; (**B**) Direct-acting antivirals vs. no intervention.

Six studies provided data on head-to-head comparisons between HCC recurrence rates after DAA vs. IFN therapy. The risk of HCC recurrence was significantly lower after DAA therapy than an IFN-based regimen (RR, 0.64; CI, 0.51−0.81), with low heterogeneity (I^2^ = 3%) (Figure 4A).

Eleven studies provided data on head-to-head comparisons between HCC recurrence rates after DAA therapy vs. no intervention. The risk of HCC recurrence was significantly lower after DAA therapy than no intervention (RR, 0.68; CI, 0.49−0.94) with high heterogeneity (I^2^ = 87.8%) (Figure 4B).

### 3.4. Study Quality and Publication Bias

Overall, the methodological quality of the included studies was judged as low, mostly due to uncontrolled designs, retrospective natures, and short follow-up lengths. A detailed representation of the study quality evaluation is reported in Table 3.

## 4. Discussion

In the present systematic review and meta-analysis, we found that about one-third of patients with a history of curative treatment for HCC undergoing DAA therapy for HCV infection experience HCC recurrence. Notably, this risk seems to be the highest early during follow-up, i.e., within 6 months from the end of DAA treatment, and among Asian countries. Furthermore, DAA therapy significantly reduces the risk of HCC recurrence compared to an IFN regimen and no intervention.

Some studies initially suggested that DAA treatment might increase the recurrence rate for HCC [8], raising concern about this therapy for patients with a history of HCC. Nevertheless, subsequent evidence did not confirm such an association, both for HCC recurrence and for HCC risk overall [16,44], leading international guidelines to eventually also recommend DAA therapy in such patients [45]. In this meta-analysis, we comprehensively showed that HCC recurrence is significantly lower after DAA therapy than an IFN-based regimen and no intervention. This result validates current guidelines and confirms and expands previous findings by Saraiya et al. [9], who showed that DAA-treated patients had a lower risk of HCC recurrence than untreated patients, while they did not compute any pooled analysis for DAA vs. IFN due to a paucity of data. On the other hand, Waziry et al. [7] found a higher HCC recurrence rate for DAA vs. IFN-treated patients, which became no longer significant after adjusting for study follow-up and patient age. Although they were not confirmatory, as they were provided by pooled study-level analysis, these findings are informative for clinicians. A possible explanation for such results lies in the decrease in active viremia induced by antiviral therapy. In fact, DAA therapy attains much higher sustained virological response (SVR) rates than IFN [45]; this might be, in turn, responsible for a decreased inflammatory stimulus and, therefore, reduce the neoplastic drive in the liver. An effect mediated through fibrosis regression and portal hypertension improvement seems to be less likely, as most of the included studies had a limited duration of follow-up, which probably prevented such modifications from happening.

Whether or not it is clear that we have to treat all patients infected by HCV with DAA, we should note that the rate of HCC recurrence was not low, being around one-third of patients, mostly within the first six months after antiviral therapy. However, these data should be taken with caution, as the mean time interval between HCC curative treatment and DAA therapy was low in some of the included studies. Therefore, it is possible that some patients already had recurrent HCC when DAA therapy was started. This would clearly lead to an overestimation of the burden of HCC recurrence.

We observed a tendency towards a significant difference in HCC recurrence rates according to the geographical region, ranging from 23% in European studies to 33% in Asian studies and 50% in the only study conducted in Australia. There are some possible explanations underlying this result. On one hand, these data might reflect differences in HCV genotype distribution, as genotype 3 has been found to increase liver fibrosis [46] and genotype 1 has been associated with an increased risk of HCC development [47]. Even though a large modelling study from the Polaris Observatory HCV Collaborators [48] found genotype 1 to be much more frequent in Asia than western Europe, paving the way to this pathophysiological theory, no stratified data on HCV genotypes were provided by the studies, preventing us from verifying this hypothesis. On the other hand, the higher HCC recurrence rates observed in Asian studies might be explained by different ethnicities’ genetic backgrounds. In fact, a cohort study conducted on more than 400 patients with cirrhosis due to HCV infection in the US found that HCC risk was increased 4-fold in Asians and doubled in African American men, compared to Caucasians [48].

Our findings expand the previously published systematic review and meta-analysis by Saraiya et al. [9] and by Waziry et al. [7], including 24 studies with 1820 patients and 10 studies with 867 patients focusing on HCC recurrence, respectively. We believe that at least three reasons for novelty can be found. First, more than twenty full-text studies were published since Saraiya and Waziry’s reviews, permitting us to achieve more consistent pooled estimates. Second, we decided to include only full-text studies, differently from Saraiya et al. [9], who also included conference abstracts. Although this choice might have partly decreased the total size of our meta-analysis, we feel that this approach was more rigorous. Third, we found in a sub-analysis that more than two-thirds of recurrent HCCs developed within six months after DAA treatment, providing insightful information to clinicians.

Our meta-analysis has strengths and limitations. On one hand, the quality of most of the included studies was low, owing to the uncontrolled designs, the retrospective natures, and the short follow-up durations after DAA therapy. Furthermore, most studies did not provide enough data on patients’ characterization, e.g., regarding the baseline HCC features, and the degree and duration of liver cirrhosis before DAA treatment. This clearly affects the degree of confidence in our estimates. On the other hand, we followed the PRISMA recommendations [10] for conducting systematic reviews and meta-analyses. Furthermore, the decision to include full-text studies only was undertaken in order to reduce the study-related bias. Although high heterogeneity affected our primary outcome, we tried to explain it through several subgroup analyses and metaregression analyses, providing useful hints for future research. Last, the significant reduction in the HCC recurrence rate for DAA-treated patients vs. IFN-treated and untreated subjects is explorative and not confirmative. Indeed, most of the included studies did not provide enough information on patients’ clinical factors potentially affecting HCC recurrence, e.g., history of previous HCC recurrence, the type of HCC treatment, the degree of liver dysfunction, and the tumor burden. Furthermore, we decided not to adjust for any study-related variables such as the mean follow-up, as the number of included studies for this comparative outcome was small.

In conclusion, we found that HCC recurrence in patients with prior HCC history after DAA therapy is not infrequent, as it concerns about one-third of patients, especially early after the end of DAA therapy. Of note, DAA therapy seems to reduce the risk of HCC recurrence compared to an IFN regimen and no intervention according to unadjusted analysis. Prospective, multicenter studies with complete information on patients’ characteristics that might affect HCC recurrence are needed.

## Figures and Tables

**Figure 5:**
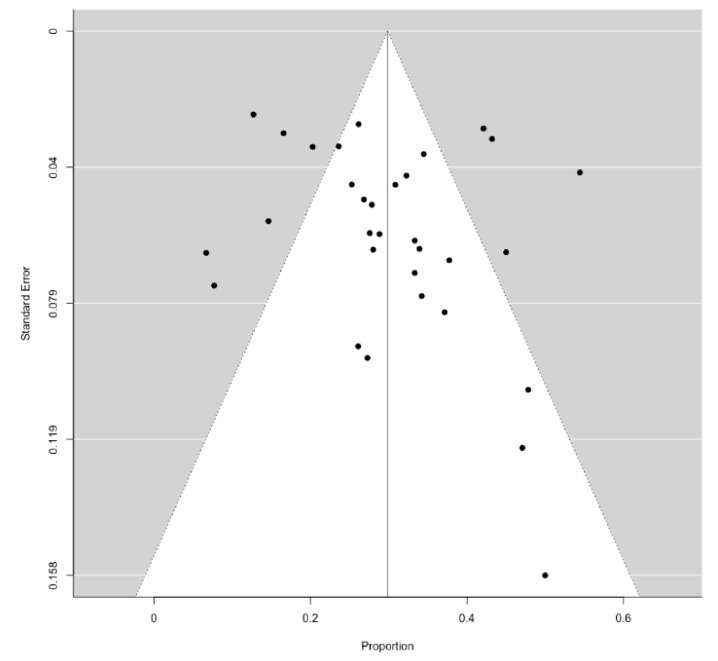
Funnel plot describing publication bias for the primary outcome.

**Table 1:** Preferred Reporting Items for Systematic Reviews and Meta-Analyses (PRISMA) checklist for the included studies.

Section/Topic	#	Checklist Item	Reported on Page
**Title**			
Title	1	Identify the report as a systematic review, meta-analysis, or both.	1
**Abstract**			
Structured summary	2	Provide a structured summary including, as applicable, background; objectives; data sources; study eligibility criteria, participants, and interventions; study appraisal and synthesis methods; results; limitations; conclusions and implications of key findings; and systematic review registration number.	1
**Introduction**			
Rationale	3	Describe the rationale for the review in the context of what is already known.	1,2
Objectives	4	Provide an explicit statement of questions being addressed with reference to participants, interventions, comparisons, outcomes, and study design (PICOS).	2
**Methods**			
Protocol and registration	5	Indicate if a review protocol exists, and if and where it can be accessed (e.g., web address), and, if available, provide registration information including registration number.	-
Eligibility criteria	6	Specify study characteristics (e.g., PICOS and length of follow-up) and report characteristics (e.g., years considered, language, and publication status) used as criteria for eligibility, giving rationale.	2–4
Information sources	7	Describe all information sources (e.g., databases with dates of coverage, contact with study authors to identify additional studies) in the search and date last searched.	2–4
Search	8	Present full electronic search strategy for at least one database, including any limits used, such that it could be repeated.	2–4
Study selection	9	State the process for selecting studies (i.e., screening, eligibility, included in systematic review, and, if applicable, included in the meta-analysis).	2–4
Data collection process	10	Describe method of data extraction from reports (e.g., piloted forms, independently, and in duplicate) and any processes for obtaining and confirming data from investigators.	2–4
Data items	11	List and define all variables for which data were sought (e.g., PICOS and funding sources) and any assumptions and simplifications made.	2–4
Risk of bias in individual studies	12	Describe methods used for assessing risk of bias of individual studies (including specification of whether this was done at the study or outcome level), and how this information is to be used in any data synthesis.	2–4
Summary measures	13	State the principal summary measures (e.g., risk ratio and difference in means).	4
Synthesis of results	14	Describe the methods of handling data and combining results of studies, if done, including measures of consistency (e.g., I^2^) for each meta-analysis.	4
Risk of bias across studies	15	Specify any assessment of risk of bias that may affect the cumulative evidence (e.g., publication bias and selective reporting within studies).	4
Additional analyses	16	Describe methods of additional analyses (e.g., sensitivity or subgroup analyses, metaregression), if done, indicating which were pre-specified.	4
**Results**			
Study selection	17	Give numbers of studies screened, assessed for eligibility, and included in the review, with reasons for exclusions at each stage, ideally with a flow diagram.	4,5, Figure 1
Study characteristics	18	For each study, present characteristics for which data were extracted (e.g., study size, PICOS, follow-up period) and provide the citations.	4,5, Table 2
Risk of bias within studies	19	Present data on risk of bias of each study and, if available, any outcome level assessment (see Item 12).	Table 3
Results of individual studies	20	For all outcomes considered (benefits or harms), present, for each study, (a) simple summary data for each intervention group, and (b) effect estimates and confidence intervals, ideally with a forest plot.	Figures 2–4
Synthesis of results	21	Present results of each meta-analysis performed, including confidence intervals and measures of consistency.	Figures 2–4
Risk of bias across studies	22	Present results of any assessment of risk of bias across studies (see Item 15).	10, Figure 5
Additional analysis	23	Give results of additional analyses, if performed (e.g., sensitivity or subgroup analyses, and metaregression (see Item 16)).	10
**Discussion**			
Summary of evidence	24	Summarize the main findings including the strength of evidence for each main outcome; consider their relevance to key groups (e.g., healthcare providers, users, and policymakers).	11
Limitations	25	Discuss limitations at study and outcome level (e.g., risk of bias), and at review level (e.g., incomplete retrieval of identified research, and reporting bias).	12
Conclusions	26	Provide a general interpretation of the results in the context of other evidence, and implications for future research.	11–13
**Funding**			
Funding	27	Describe sources of funding for the systematic review and other support (e.g., supply of data), and role of funders for the systematic review.	13

From: Moher, D.; Liberati, A.; Tetzlaff, J.; Altman D.G., The PRISMA Group (2009). Preferred Reporting Items for Systematic Reviews and Meta-Analyses: The PRISMA Statement. PLoS Med 6(6): e1000097. doi:10.1371/journal.pmed1000097. For more information, visit www.peisma-statement.org.

**Table 2:** Characteristics of included studies reporting hepatocellular carcinoma (HCC) recurrence after direct-acting antiviral (DAA) therapy.

Reference	Year	Study Design	Country	*n* Patients Treated with DAA	Mean Time from HCC Treatment to DAA (Months)	Mean Follow-Up (Months)
Bielen [15]	2017	retrospective	Belgium	41	33	6
Cabibbo [16]	2017	prospective	Italy	143	1.8	9.1
Ikeda [21]	2017	retrospective	Japan	177	20.1	20.4
Nagata [30]	2017	retrospective	Japan	79		22.3
Ogawa [33]	2017	prospective	Japan	157	35.2	16.6
Reig [36]	2016	retrospective	Spain	58	12.8	6
Virlogeux [41]	2017	retrospective	France	23	13	12
Conti [8]	2016	retrospective	Italy	59	17.6	5.5
ANRS [14] (CO22 HEPATHER)	2016	retrospective	France	189	19.2	20.2
ANRS [14] (CO12 Cirvir)	2016	retrospective	France	13		
Rinaldi [37]	2016	retrospective	Italy	15	11.3	3
El Kassas [20]	2018	prospective	Egypt	53		
Ooka [34]	2018	prospective	Japan	95		7.4
Lashen [24]	2019	retrospective	Egypt	50	5.18	15
Lleo [27]	2019	retrospective	Italy	161	12	
Preda [35]	2019	prospective	Romania	22	27.7	49.7
Nishibatake Kinoshita [31]	2019	retrospective	Japan	147	7.1	1.8
Kogiso [22]	2019	retrospective	Japan	45	36.1	25.2
Yoshimasu [42]	2019	retrospective	Japan	23		16.5
Nagaoki [29]	2019	retrospective	Japan	38	35.2	
Degasperi [19]	2019	retrospective	Italy	60	24.9	
Singal [39]	2019	retrospective	USA and Canada	304	6.8	12.3
Zou [43]	2019	retrospective	USA	264	30.9	23.3
Chi [18]	2019	retrospective	Taiwan	107	14.5	32.3
Kuo [23]	2019	retrospective	Taiwan	82	30.7	
Chan [17]	2020	retrospective	Australia	10	18.3	23.8
Lin [25]	2020	retrospective	Taiwan	35	29.3	18.9
Miuma [28]	2020	retrospective	Japan	17	26.4	
Sangiovanni [38]	2020	prospective	Italy	124	20.2	15.1
Tahata [40]	2020	retrospective	Japan	250	21.4	27
Lithy [26]	2020	prospective	Egypt	60	12	
Ochi [32]	2020	retrospective	Japan	56	5.7	39.9

**Table 3:** Methodological quality of included studies according to Newcastle–Ottawa scale. *, low risk of bias.

Reference	Representative Cohort	Ascertainment of Exposure	Outcome Not Present at Start	Outcome Assessment	Follow-Up Period	Follow-Up Adequacy
Bielen [15]	*	*	High	High	*	High
Cabibbo [16]	*	*	*	*	High	Unknown
Ikeda [21]	*	*	Medium	Medium	*	*
Nagata [30]	*	*	High	Medium	*	Unknown
Ogawa [33]	*	*	*	*	*	Unknown
Reig [36]	*	*	*	Medium	High	*
Virlogeux [41]	*	*	*	Medium	*	High
Conti [8]	*	*	Medium	Medium	High	*
ANRS [14] (CO22 HEPATHER)	*	*	Medium	High	*	Unknown
ANRS [14] (CO12 Cirvir)	*	*	Medium	High	*	Unknown
Rinaldi [37]	*	*	*	Medium	High	Unknown
El Kassas [20]	*	*	High	High	Unknown	Unknown
Ooka [34]	*	*	High	High	Medium	High
Lashen [24]	*	*	High	*	*	*
Lleo [27]	*	*	*	High	*	Medium
Preda [35]	High	*	*	High	*	High
Nishibatake Kinoshita [31]	*	*	Medium	High	High	*
Kogiso [22]	*	*	*	High	*	Unknown
Yoshimasu [42]	High	*	High	High	*	*
Nagaoki [29]	*	*	*	Medium	*	*
Degasperi [19]	*	*	High	High	*	*
Singal [39]	*	*	Medium	*	*	Unknown
Zou [43]	*	*	*	*	*	High
Chi [18]	*	*	*	*	*	*
Kuo [23]	*	*	*	*	Unknown	*
Chan [17]	High	*	*	High	*	High
Lin [25]	*	*	*	*	*	*
Miuma [28]	High	*	*	High	Unknown	Unknown
Sangiovanni [38]	*	*	*	*	*	*
Tahata [40]	*	*	*	Medium	*	Medium
Lithy [26]	*	*	High	High	*	*
Ochi [32]	*	*	Unknown	High	*	Medium

No significant publication bias was detected for the primary outcome (i.e., the HCC recurrence during follow-up), according to both a visual inspection of the funnel plot (Figure 5) and regression test (*p* = 0.198).

## Abbreviations

CI	confidence interval
DAA	direct-acting antivirals
HCC	hepatocellular carcinoma
HCV	hepatitis C virus
PEG-IFN	pegylated interferon
PRISMA	Preferred Reporting Items for Systematic Review and Meta-analysis
SVR	sustained virological response
